# Supplementary material for: Intrinsic Shapes of Empathy: Functional Brain Network Topology Encodes Intersubjective Experience and Awareness Traits
Source: Brain Sci. 2022 Apr 5;12(4):477. doi: 10.3390/brainsci12040477 (PMC9024660; doi:10.3390/brainsci12040477)
Supplement: Supplementary file 1 [file brainsci-12-00477-s001.zip › brainsci-1650311-supplementary.pdf]

SUPPLEMENTARY MATERIAL

Intrinsic shapes of empathy: functional brain network topology encodes intersubjective experience and awareness traits

By

Sjoerd J.H. Ebisch, Andrea Scalabrini, Georg Northoff, Clara Mucci, Maria Rita Sergi, Aristide Saggino, Antonio Aquino, Francesca R. Alparone, Mauro Gianni Perrucci, Vittorio Gallese, Simone Di Plinio

**Degree Centrality & Vicarious Experience**	**X**	**Y**	**Z**	**β**
Whole Module	**-**	**-**	**-**	***3.2***
**G_Frontal_Mid-2-L**	-32	44	25	6.37
**S_Orbital-1-L**	-25	38	-15	6.06
**G_SupraMarginal-5-R**	60	-41	28	5.77
**G_Frontal_Mid-2-R**	32	45	27	5.72
**G_Frontal_Mid-3-R**	38	34	35	5.33
**G_Frontal_Mid-1-L**	-40	41	20	5.05
**S_Intraparietal-2-L**	-34	-58	45	4.89
**G_SupraMarginal-6-L**	-53	-43	44	4.88
**S_Sup_Frontal-2-L**	-27	55	0	4.84
**S_Sup_Frontal-2-R**	28	57	7	4.63
**G_Temporal_Inf-3-L**	-56	-53	-14	4.44
**G_Parietal_Inf-1-L**	-45	-53	48	4.30
**G_Precuneus-9-R**	13	-68	49	4.27
**G_Insula-anterior-4-L**	-41	15	2	4.25
**S_Sup_Frontal-3-L**	-25	43	29	4.15
**G_SupraMarginal-6-R**	54	-38	43	4.13
**G_Temporal_Mid-4-L**	-53	-59	6	4.06
**S_Inf_Frontal-1-R**	47	39	10	4.06
**G_Frontal_Sup_Medial-3-R**	6	33	44	4.00
**G_Frontal_Mid-3-L**	-38	31	35	3.97
**G_Parietal_Inf-1-R**	43	-53	47	3.80
**G_Frontal_Mid-4-R**	39	21	46	3.76
**G_Precuneus-6-L**	-8	-62	61	3.73
**S_Precentral-1-R**	51	9	24	3.73
**G_Supp_Motor_Area-1-R**	6	20	48	3.40
**S_Inf_Frontal-2-L**	-43	15	29	3.39
**G_Frontal_Mid_Orb-2-L**	-41	49	-5	3.32
**G_Supp_Motor_Area-1-L**	-7	22	45	3.30

Table S1. Details of regional associations between degree of centrality and vicarious experience (refer to Figure 3a). Only the strongest associations are shown for readability (S=Sulcus, G=Gyrus, C=Cerebellar node, N=Nucleus; L=Left, R=Right).

**Betweenness Centrality & Vicarious Experience**	**X**	**Y**	**Z**	**β_{FEM}**	**β_{MAL}**
Whole Module	***-***	***-***	***-***	***82***	***-19***
**S_Cingulate-2-L**	-7	16	40	164.666	-15.93
**S_Cingulate-1-R**	8	27	31	163.978	-44.295
**G_Supp_Motor_Area-1-L**	-7	22	45	150.966	-91.021
**G_Supp_Motor_Area-1-R**	6	20	48	147.406	-122.575
**G_Cingulum_Mid-1-R**	4	15	28	144.716	14.793
**S_Cingulate-2-R**	8	14	45	143.621	34.828
**G_Supp_Motor_Area-3-R**	7	10	65	141.691	39.714
**G_Temporal_Mid-4-L**	-53	-59	6	123.394	-13.279
**G_Angular-1-R**	51	-52	42	113.474	-22.57
**G_Insula-anterior-3-R**	37	24	0	109.508	30.738
**G_Frontal_Inf_Orb-2-L**	-21	23	-22	104.859	-63.885
**S_Sup_Frontal-2-R**	28	57	7	102.89	-53.39
**G_Frontal_Mid-2-R**	32	45	27	101.378	-101.833
**S_Intraparietal-3-L**	-27	-60	43	100.91	-94.149
**G_Precuneus-9-R**	13	-68	49	100.399	-31.064
**G_Frontal_Sup_Medial-3-R**	6	33	44	99.329	-62.827
**S_Sup_Frontal-3-L**	-25	43	29	93.809	-58.721
**G_Frontal_Mid-4-R**	39	21	46	88.947	-102.085
**S_Intraparietal-2-R**	37	-53	48	86.073	-51.289
**G_SupraMarginal-5-R**	60	-41	28	85.401	-27.351

Table S2. Details of regional associations between betweenness centrality and vicarious experience for females and males (refer to Figure 3b). Only the strongest associations are shown for readability (S=Sulcus, G=Gyrus, C=Cerebellar node, N=Nucleus; L=Left, R=Right).

**Participation Coefficient & Vicarious Experience**	**X**	**Y**	**Z**	**β**
Whole Module	***-***	***-***	***-***	***.016***
**S_Orbital-1-L**	-25	38	-15	0.026
**G_Cingulum_Mid-2-L**	-4	4	29	0.025
**G_Frontal_Inf_Orb-2-L**	-21	23	-22	0.022
**S_Sup_Frontal-1-L**	-21	62	-8	0.020
**S_Orbital-1-R**	25	42	-15	0.019
**G_Frontal_Mid-1-R**	42	45	13	0.018
**G_Cingulum_Mid-2-R**	4	4	29	0.018
**S_Orbital-2-L**	-31	34	-13	0.017
**S_Sup_Frontal-1-R**	20	63	-6	0.017
**G_SupraMarginal-5-R**	60	-41	28	0.016

Table S3. Details of regional associations between participation coefficient and vicarious experience (refer to Figure 3c). Only the strongest associations are shown for readability (S=Sulcus, G=Gyrus, C=Cerebellar node, N=Nucleus; L=Left, R=Right).

**Degree Centrality & Intuitive Understanding**	**X**	**Y**	**Z**	**β_{FEM}**	**β_{MAL}**
Whole Module	***-***	***-***	***-***	***2.9***	***-0.3***
**N_Caudate-3-L**	-12	14	-8	4.40	-0.84
**N_Caudate-1-L**	-11	22	1	3.93	-0.04
**N_Caudate-4-L**	-15	11	12	3.63	-0.20
**N_Putamen-3-R**	29	-4	1	2.73	-0.03
**N_Putamen-2-R**	23	8	0	2.51	-0.68
**N_Caudate-6-L**	-16	6	18	2.39	-0.36
**N_Caudate-4-R**	13	20	8	2.26	-0.31
**N_Caudate-6-R**	15	7	17	2.13	-0.92
**N_Putamen-3-L**	-27	-6	2	2.12	-0.14
**N_Putamen-2-L**	-23	6	0	1.99	-0.49
**N_Caudate-2-L**	-15	27	-1	1.95	0.08
**N_Caudate-5-R**	12	10	9	1.94	-0.22

Table S4. Details of regional associations between degree of centrality and intuitive understanding for females and males (refer to Figure 4a). Only the strongest associations are shown for readability (S=Sulcus, G=Gyrus, C=Cerebellar node, N=Nucleus; L=Left, R=Right).

Betweenness Centrality & Intuitive Understanding	X	Y	Z	β
Whole Module	**-**	**-**	**-**	***25***
**G_Supp_Motor_Area-3-R**	7	10	65	187.55
**S_Cingulate-6-L**	-11	-35	46	178.13
**S_Cingulate-6-R**	12	-34	45	153.39
**S_Cingulate-5-R**	9	-15	41	120.50
**S_Cingulate-7-R**	10	-41	60	93.59
**S_Sup_Frontal-6-R**	24	2	58	92.64
**S_Cingulate-7-L**	-9	-42	59	89.43
**S_Cingulate-1-R**	8	27	31	80.35
**C_Left_VIIb**	-25	-67	-51	77.64
**N_Putamen-3-R**	29	-4	1	75.44
**G_Paracentral_Lobule-3-R**	8	-26	70	74.10
**S_Cingulate-3-R**	8	5	51	70.93
**G_Paracentral_Lobule-4-L**	-7	-30	75	69.04
**G_Temporal_Mid-4-R**	57	-54	3	67.17
**G_SupraMarginal-5-L**	-57	-43	29	63.11
**S_Precentral-2-L**	-25	-9	58	62.52
**S_Rolando-2-R**	46	-11	49	62.07
**S_Rolando-3-L**	-38	-23	60	61.92
**S_Rolando-3-R**	37	-20	60	60.83
**N_Thalamus-5-R**	13	-18	6	57.21
**G_Temporal_Sup-4-L**	-58	-23	3	54.46

Table S5. Details of regional associations between betweenness centrality and intuitive understanding (refer to Figure 4b). Only the strongest associations are shown for readability (S=Sulcus, G=Gyrus, C=Cerebellar node, N=Nucleus; L=Left, R=Right).

**Participation Coefficient & Intuitive Understanding**	**X**	**Y**	**Z**	**β_{FEM}**	**β_{MAL}**
Whole Module	**-**	**-**	**-**	***.029***	***-.001***
**G_Supp_Motor_Area-3-L**	-7	8	63	0.023	-0.007
**G_SupraMarginal-5-R**	60	-41	28	0.020	-0.007
**G_Supp_Motor_Area-3-R**	7	10	65	0.019	-0.003
**G_Insula-anterior-4-L**	-41	15	2	0.018	0.001
**S_Precentral-1-R**	51	9	24	0.015	-0.004
**G_Frontal_Mid-2-L**	-32	44	25	0.014	-0.004
**S_Cingulate-1-R**	8	27	31	0.013	-0.003
**S_Cingulate-1-L**	-7	27	30	0.011	0.001

Table S6. Details of regional associations between participation coefficient and intuitive understanding for females and males (refer to Figure 4c). Only the strongest associations are shown for readability (S=Sulcus, G=Gyrus, C=Cerebellar node, N=Nucleus; L=Left, R=Right).
